# Editorial: Bio-Inspired Physiological Signal(s) and Medical Image(s) Neural Processing Systems Based on Deep Learning and Mathematical Modeling for Implementing Bio-Engineering Applications in Medical and Industrial Fields

**DOI:** 10.3389/fninf.2021.763699

**Published:** 2021-10-29

**Authors:** Francesco Rundo, Giuseppe Luigi Banna, Concetto Spampinato, Sabrina Conoci

**Affiliations:** ^1^STMicroelectronics, ADG Central R&D, Catania, Italy; ^2^United Lincolnshire Hospitals NHS Trust, Louth, United Kingdom; ^3^PerCeiVe Lab, University of Catania, Catania, Italy; ^4^Department of Chemical, Biological, Pharmaceutical and Environmental Sciences, University of Messina, Messina, Italy

**Keywords:** neural computing, bio-inspired systems, bio-engineering, industrial applications, automotive

This Research Topic gathers different contributions highlighting novel types of bio-inspired mathematical models with neuroimaging and physio-signal data, mainly applied in medical and industrial field. The target of this Research Topic was to collect scientific contributions which were able to highlight the undoubted advantages that modern techniques of Deep Learning and Bio-inspired Neural computing can offer in addressing the main issues in the medical and industrial field. The correlated target of the Research Topic was also to highlight the significant contribution of multimodal data analysis (signals and images) in the medical and industrial field. The collected accepted articles contribute significantly to the achievement of the Research Topic target, confirming, as below detailed, the capabilities of the combined approach of bio-inspired mathematical models and multimodal data analysis.

The first accepted article of this Research Topic (Folego et al.) proposed an early detection method of Alzheimer's disease (AD) by means of a 3D Convolutional Deep Network applied to MRI imaging of the analyzed subjects. It is well-known that early diagnosis is paramount to the development and success of interventions, and neuroimaging represents one of the most promising areas for early detection of AD. Through the neuroimaging deep analysis the authors investigated the implementation of such biomarkers suitable to classify brain images into AD, mild cognitive impairment (MCI), and cognitively normal (CN) groups. Their deep network solution embedded domain adaptation to improve the performance in neuro-imaging classification. The performance of the proposed solution shows promising results for CN discrimination (67.3% with TPF metric) against lower performance for MCI and AD early detection. Anyway, the collected results outperformed the compared similar pipelines.

Still in the medical field, the contribution proposed by Zhen et al. is of considerable interest. The authors proposed a deep learning-based pipeline for accurate diagnosis of a Liver cancer based on the analysis of multimodal data i.e., MRI imaging combined with such clinical data of the analyzed patients. Several scientific contributions confirmed that early-stage diagnosis and treatment of liver cancer can improve survival rates. Dynamic contrast-enhanced MRI provides the most comprehensive information for differential diagnosis of liver tumors. However, MRI-based diagnosis is affected by subjective physicians experience in addition to the difficulties implicit in the method (limited spatial resolution). The authors showed that deep learning solutions they implemented may supply to these limitations. To improve the performance of the developed deep architecture, the authors processed MRI images combined with clinical data. They analyzed a dataset of 1,210 patients with liver tumors (*N* = 31,608 images) used as training set while the learned model were validated in an external independent extended cohort of 201 patients (*N* = 6,816 images). Using only unenhanced images, the proposed deep classifier performs well in distinguishing malignant from benign liver tumors (AUC, 0.946; 95% CI 0.914–0.979 vs. 0.951; 0.919–0.982, *P* = 0.664). The authors tried to combine unenhanced MRI images with clinical data. This setup improved significantly the performance of classifying malignancies as hepatocellular carcinoma (AUC, 0.985; 95% CI 0.960–1.000), metastatic tumors (0.998; 0.989–1.000), and other primary malignancies (0.963; 0.896–1.000). The very promising results confirmed that multimodal data processing through *ad-hoc* deep classifier can be considered as valuable tool for robust differential diagnosis of liver cancer.

In Henriques-Pons et al. the authors proposed an interesting mathematical analysis to the flow cytometry labeling. Conventional flow cytometry analysis relies on the creation of dot plot sequences, based on two fluorescence parameters at a time, to evidence phenotypically distinct populations. Anyway, results of such classical approach is not always robust. The authors proposed an interesting mathematical analysis named MCTA (Multiparametric Color Tendency Analysis) which considers multiple labelings simultaneously, extending and complementing conventional analysis. The MCTA method executes the background fluorescence exclusion, spillover compensation, and a user-defined gating strategy for subpopulation analysis. The performance showed are very promising and then the MCTA it deserves to be further investigated for applications in the biomolecular field.

Still in medical oncology field, an interesting literature review was proposed by Sa et al. in which the Fluoro-Deoxyglucose Positron Emission Tomography/Computed Tomography (PET/CT) imaging method was analyzed for Pediatric Rhabdomyosarcoma (RMS) staging and prognosis estimation. Thirteen consecutive patients with pathologically confirmed RMS underwent PET/CT scan for evaluation of therapy response. About the baseline PET/CT, most RMSs are located in the pelvic cavity, and upper arms ranked second. About evaluation of the disease spread, lymph node metastases were seen in eight patients, and eight patients showed distant metastasis to the lung, liver, and bone. The median SUVmax, SUVmean, and SUVpeak of primary sites were 7.1, 4.0, and 5.9, respectively. The scientific paper confirmed that PET/CT scan methodology could be a valid tool for RMS assessing as this kind of cancer demonstrates increased glycolytic activity. Again, *ad-hoc* mathematical analysis of imaging data showed very promising results in medical field.

A very interesting approach in the field of neurology was proposed by Li et al. in which the authors implemented a mathematical approach for distinguishing Epileptiform Discharges (ED) from normal electroencephalograms (EEG). It is well-known that EDs are of fundamental importance in understanding the physiology of epilepsy. To aid in the clinical diagnosis, classification, prognosis, and treatment of epilepsy, it is important to implement robust solutions to distinguish epileptiform discharges from normal electroencephalogram (EEG). This is a challenging task. To take on this challenge, they proposed to use a multiscale complexity measure, the scale-dependent Lyapunov exponent (SDLE). The authors analyzed 640 multi-channel EEG segments, each 4 s long. Among these segments, 540 are short epileptiform discharges, and 100 are from healthy controls. They noticed that such SDLE features can be effective in distinguishing epileptiform discharges from normal EEG. They tested different machine learning classifier such as Random Forest Classifier (RF) and Support Vector Machines (SVM), obtaining an accuracy around 99% in distinguishing ED from normal EEG. The confirmed robustness of the approach based on SDLE analysis suggest further investigation of the approach with the target to use that widely in a clinical setting.

Another promising pipeline based on multimodal data analysis for applications in the medical field can be found in the contribution (Bartoletti et al.) in which the authors correlated such clinical data of the patients with Bioelectric Impedance Analysis (BIA) in order to select the patients candidate to prostate biopsy. The authors analyzed a cohort of one-hundred 40 consecutive candidates to prostate biopsy and 40 healthy volunteers. For each recruited subject the following clinical data have been collected: PSA and PSA density determinations (PSAD), Digital Rectal Examination (DRE), and the novel BIA test. The targets of the proposed investigation were to determine accuracy of BIA test in comparison to PSA, PSAD levels and MRI and obtain Prostate Cancer (PCa) prediction by BIA test. The authors performed a lot of experimental results which confirmed what follow: Combined PSA, PSAD, DRE, and trans rectal ultrasound test failed to discern patients with PCa from those with benign disease (62.86% accuracy; sensitivity of 83% and a specificity of 59%). The accuracy in discerning PCa increased up to 75% by BIA test (sensitivity 63.33% and specificity 83.75%) confirming the effectiveness of the proposed approach.

Another field of application of the analyzed Research Topic was the investigation of bio-inspired models and solutions. This includes the study proposed by Jia et al. which analyzed the impact of some Neuron Dendritic Spine Patterns. Some pattern abnormalities of dendritic spine, seems to be correlated to multiple nervous system diseases, such as Parkinson's disease and schizophrenia. The analysis of these spine patterns can help to bring-up a valid model of the pathogenesis of these diseases. The authors investigated the application of the bio-inspired reaction-diffusion model to simulate the formation patterns dynamic of dendritic spine. The authors also investigated the deep regulation mechanisms of dendritic spine. The authors was able to define a robust mathematic model-based pathogenesis research for neuron diseases correlated to the dendritic spine pattern abnormalities analysis. With this bio-inspired model, it will be possible to better study the pathogenesis of certain neurodegenerative diseases in order to identify an effective treatment.

The field of medical oncology has been significantly explored in the Research Topic. The authors of the contribution reported in Gao et al. have invested the application of modern machine learning methods to the assessment of the tumor grading and such bio-marker (Ki67, GFAP expression level, S100 expression level, etc.) of such type of brain cancer (Gliomas). The grading and pathologic biomarkers play a key role in the diagnosis and treatment of the Glioma. The authors proposed a pipeline aimed to use conventional machine learning algorithms to predict the tumor grades and pathologic biomarkers based on the analysis of magnetic resonance imaging (MRI) data. The authors analyzed a dataset of 367 glioma patients, who had pathological reports and underwent MRI imaging scans. The extracted MRI image features was processed by several type of machine learning based classifier such as Logistic Regression based classifier (LR), Support Vector Machine (SVM), and Random Forests (RF). The RF algorithm outperformed the compared classifiers i.e., Logistic Regression and SVM. The RF classifier on glioma grades achieved a predictive performance (AUC: 0.79, accuracy: 0.81) and a predictive performance of AUC: 0.85, accuracy: 0.80 for the Ki67 expression AUC: 0.72 and accuracy: 0.81 for the GFAP and AUC: 0.60 accuracy: 0.91 for the S100 expression level. The robustness of this results encourages further investigation in this area.

In the field of neurovascular diagnostics, multimodal data analysis showed significant contribution. The authors of the contribution (Zhang et al.) investigated the application of PLA-combined ferroferric graphene oxide aspirin (Fe_3_O_4_-GO-ASA) multifunctional nanobubbles in the prevention and treatment of thrombotic events in a preclinical study. The experimental results confirmed that PLA-combined Fe_3_O_4_-GO-ASA nanobubbles treatment has significantly inhibit thrombosis (concentration of 80 mg·mL−1 interacted with the rabbit blood). The proposed approach also showed a relevant ultrasonic imaging effect and a good magnetic targeting for an efficient diagnostic of the vascular disease.

The multi-modal bio-medical data analysis also involved the study of the motility and shape of the tumor cells of a subject to characterize the biology of the tumor and the selection of the most appropriate treatment. The authors of the study reported in D'Orazio et al. analyzed motility and such shape features of cancer cells *in vitro* in order to assess diagnosis and treatment. They combined fluorescence time-lapse microscopy (TLM) and label-free imaging, with cell tracking, quantitative representation of cell trajectories, and a recent machine learning (ML) strategies based on peer prediction algorithm. The implemented *ad-hoc* cooperative learning approaches in order to discriminate with high accuracy non-cancer vs. cancer cells of high vs. low malignancy. They investigated the performance of the proposed solution in the treatment of prostate cancer. Comparison with standard classification methods validated their promising proposed approach.

Still with reference to prostate cancer, a pre-clinical study reported in Merisaari et al. analyzed the impact of the mathematical modeling of MRI-DWI (Diffusion Weighted Imaging) sequences in prostate cancer assessment. Classical approaches assess the tumor growth by weekly examination of the DWI imaging by using a 7T MRI scanner. The authors implemented *ad-hoc* mathematical models for performing additional DWI examination. They observed significant changes in their DWI data mathematical features (stretched exponential and kurtosis) during the tumor growth confirming that the proposed approach shows promising performance as a robust tool for the prostate cancer follow-up.

In the field of medical neurology, an interesting multi-modal approach was presented by the authors of the contribution reported in Chen et al. They investigated the performance of such machine learnings approaches for seizure prediction based on the analysis of EEG data. They implemented a multi-dimensional enhanced seizure prediction framework, which embedded a graph state encoder, and a space-time predictor. The input data was the multi-channels EEG. Their proposed model analyzed the space-time relationship of the input data with the seizure event in epileptic subject. The authors validated their approach on a public dataset retrieving a sensitivity of 98.61%, confirming the effectiveness of the proposed solution.

Unfortunately, to date, there are particularly aggressive tumors that unfortunately denote an high rate of mortality. Among these it is worth mentioning the pancreatic cancer which in some forms is particularly lethal. The authors of the contribution reported in Han et al. investigated the reliability of radiomics pipeline applied to contrast-enhanced CT scan imaging data for discriminating pancreatic cystadenomas from pancreatic neuroendocrine tumors (PNETs). They implemented effective machine-learning pipelines to perform this discrimination. They retrospectively analyzed 120 patients, including 66 pancreatic cystadenomas patients and 54 PNETs patients. They identified 48 had-crafted radiomic features from contrast-enhanced CT images to be classified by classical machine learning methods such as linear discriminant analysis (LDA), Random Forest, Adaboost, support vector machine (SVM), k-nearest neighbor (KNN), logistic regression (LR), and so on. The proposed deep classifier shows promising ability of differentiating pancreatic cystadenomas from PNETs. Specifically, the RF-based classifier, as well as Xgboost+RF, demonstrated the best discriminative ability, with the highest AUC of 0.997 in the testing group.

In the context of the bio-inspired models' study (one of the main target of the Research Topics), the contribution reported in Quan et al. shows considerable interest. The authors proposed *FusionNet* a novel deep fully residual convolutional neural network for image segmentation in connectomics. Neuro-connectomics tries to generate comprehensive brain connectivity maps using high-throughput, nano-scale electron microscopy. Deep learning showed very promising performance in image processing and computer vision, leading to a recent explosion in popularity. For these reasons the author implemented *ad-hoc* deep architecture named *FusionNet* to perform automatic segmentation of neuronal structures in connectomics data. FusionNet is a fully convolutional deep architecture which embeds semantic segmentation ability combined with residual layers which improve the overall segmentation capability. The authors successfully validated their deep backbone providing robust comparison with other popular electron microscopy and confirming the very promising performance of the proposed architecture.

The analyzed scientific contributions confirmed that the convolutional architectures show remarkable skills in image processing and computer vision tasks in medical and industrial fields. In the contribution reported in Tang et al. the authors investigated the use of specific convolutional architecture for the diagnosis of Intramucosal Gastric Cancer. A deep convolutional neural network was implemented to learn a retrospectively collected 3,407 endoscopic images from 666 gastric cancer patients. The deep network performance was validated over a test-set composed by 228 images from 62 independent patients. The implemented deep architecture was able to discriminate intramucosal Gastric Cancer from advanced Gastric Cancer with an AUC of 0.942, a sensitivity of 90.5%, and a specificity of 85.3% in the testing dataset. The reported results confirmed that deep learning can be effectively used to improve diagnostic accuracy in medical oncology.

Medical branch related to oncology is certainly pathological anatomy. Also in this context, multimodal analysis together with machine learning methods can significantly improve the histopathological characterization of tumors. More in detail, in the article reported in Hao et al. the authors proposed a low dimensional three-channel features based breast cancer histopathological images classification approach suitable to discriminate benign breast cancer from malignant ones. Several hand-crafted image descriptors including gray level co-occurrence matrix on different directions, average pixel value of each channel, Hu invariant moment (HIM), wavelet features, and son on, are defined as input of Support Vector Machine classifier. Experiments on specific dataset showed that the proposed pipeline achieved an accuracy of 90.2–94.97% at the image level and 89.18–94.24% at the patient level, which outperforms many state-of-the-art methods.

As the analyzed Research Topic covered applications in the industrial field, it is of particular interest to include among the publications of this scientific issues, the contribution reported in Rundo et al. The authors investigated the usage of such neuro-physiological signals (specifically the PhotoPlethysmoGraphy) in the automotive field. With the aim to develop an intelligent driving assistance system, the authors implemented a complex system (both hardware and software) which embeds different deep architectures with innovative attention mechanisms. Specifically, the proposed system detects and tracks the car driver attention level from analysis of correlated PhotoPlethysmoGraphy sampled from a bio-sensor embedded in the car steering. This retrieved attention assessment was correlated to driving scenario risk evaluation made by another deep architecture which embeds modern self-attention mechanisms. A combined intelligent monitor evaluates the congruence between the driver's level of attention as above determined with the driving scenario risk level, generating such alerts if there is an inconsistency between the two assessments. The approach was applied in a specific automotive use-case, namely the tracking of pedestrians in a common driving scenario. In addition, further computer vision systems will assist the driver's attention assessment. The high accuracy of the proposed driver's attention detection system based on physiological analysis (more than 95%) as well as of the parallel driving risk assessment systems confirmed the effectiveness of the proposed method and of the underlying hardware platform. Therefore, also in industrial (automotive) field, the multimodal data analysis combined with bio-inspired deep processing showed high capability in addressing typical issues.

In conclusion of this scientific review, the authors hope that the reader will find in this Research Topic a useful reference for the state of the art in the emerging field of bio-inspired deep models and multimodal data analysis for addressing issues and problems in the medical and industrial field.

## Author Contributions

All authors contributed to the article and approved the submitted version.

## Conflict of Interest

FR was employed by the company STMicroelectronics. The remaining authors declare that the research was conducted in the absence of any commercial or financial relationships that could be construed as a potential conflict of interest.

## Publisher's Note

All claims expressed in this article are solely those of the authors and do not necessarily represent those of their affiliated organizations, or those of the publisher, the editors and the reviewers. Any product that may be evaluated in this article, or claim that may be made by its manufacturer, is not guaranteed or endorsed by the publisher.

